# Clustered ARPE-19 cells distinct in mitochondrial membrane potential may play a pivotal role in cell differentiation

**DOI:** 10.1038/s41598-024-73145-w

**Published:** 2024-09-27

**Authors:** Takafumi Miyatani, Hiroshi Tanaka, Kosaku Numa, Asako Uehara, Yohei Otsuki, Junji Hamuro, Shigeru Kinoshita, Chie Sotozono

**Affiliations:** 1 Department of Ophthalmology, Kyoto Prefectural University of Medicine, 465 Kajii-cho, Hirokoji-agaru, Kawaramachi-dori, Kamigyo-ku, Kyoto, 602-0841 Japan; 2 Department of Frontier Medical Science and Technology for Ophthalmology, Kyoto Prefectural University of Medicine, 465 Kajii-cho, Hirokoji-agaru, Kawaramachi-dori, Kamigyo-ku, Kyoto, 602-0841 Japan

**Keywords:** Retinal pigment epithelial (RPE) cell differentiation, Cell cluster formation, Mitochondrial membrane potential (MMP), Electron transport chain (ETC) complex I inhibitor, Eye diseases, Mitochondria

## Abstract

Age-related macular degeneration (AMD) is associated with the dysfunction and degeneration of retinal pigment epithelium (RPE) cells. Here, we examined how the formation and expansions of cell clusters are regulated by the differentiation of the RPE　cells. In this study, ARPE-19 cells were cultivated in standard or differentiation media, i.e., without or with nicotinamide, to evaluate the spreading of cell clusters specified with differentiated cell phenotypes. Mitochondria membrane potential (MMP) and the distribution of the RPE cell clusters was also monitored with or without rotenone, a mitochondrial electron transport chain (ETC) complex I inhibitor. Cultured ARPE-19 cells generated scattered cell clusters composed mostly of smaller size cells expressing the differentiation markers mouse anti-cellular retinaldehyde–binding protein (CRALBP) and Bestrophin only in differentiation medium. After the increase of the number of clusters, the clusters appeared to paracellularly merge, resulting in expansion of the area occupied by the clusters. Of note, the cells within the clusters selectively had high MMP and were in accordance with the expression of RPE differentiation markers. Rotenone repressed the formation of the clusters and decreased intracellular MMP. The above results suggest that clustering of RPE cells with functional mitochondria plays a pivotal role in RPE cell differentiation process and the ETC complex I inhibition greatly influences the composition of RPE cells that are degenerated or differentiation disposed.

**Supplementary Information:**

The online version contains supplementary material available at 10.1038/s41598-024-73145-w.

## Introduction

Age-related macular degeneration (AMD) is the leading cause of visual impairment in the elderly in developed countries and is classified into two forms, neovascular (wet) and non-neovascular (dry). In both types, age-related changes and inflammation of the retinal pigment epithelium (RPE) are responsible for the development of AMD pathogenesis, followed by photoreceptor cell death and vision loss^1–4^. The pathogenesis of AMD is thought to be multifactorial, involving a complex interaction of metabolic^5^, genetic^6,7^, epigenetic^8,9^, and environmental factors^10,11^. To maintain the differentiated phenotypes of RPE cells is critical for the functional integrity of RPE. Human RPE (hRPE) is composed of highly differentiated cells, expressing various differentiation markers such as mouse anti-cellular retinaldehyde–binding protein (CRALBP), Bestrophin, and RPE65, and only the differentiated RPE cells depict the functions of secreting cytokines and growth factors, forming a barrier, phagocytosing damaged photoreceptor outer segments (POS), and exhibiting cellular polarity and resistance to oxidative stress^12–16^.

Several previous studies have reported the convoluted culture methods to promote the differentiation of hRPE cells in vitro^17–19^. ARPE-19 cells (i.e., cells of a spontaneously arising hRPE cell line derived in 1986^15^) lose the characteristics of differentiated primary hRPE cell phenotypes after repeated cell divisions during cultures, showing the characteristics of epithelial-mesenchymal transition (EMT), and senescence phenotypes, positive for α-smooth muscle actin, p16, and p21. Hazim et al.^17^ reported an excellent method to promote the differentiation of RPE cells by culturing in the presence of nicotinamide (NAM), a method subsequently confirmed effective by our recentfindings^20^. A link between the mitochondrial metabolism of hRPE cells and its state of differentiation as a polarized epithelium has been suggested, and findings revealed that mitochondrial metabolism is a driver of the differentiation of hRPE cells into a polarized epithelium^20,21^, a conclusion partly based on the observation by Hazim et al.^21^ using UK5099 as a mitochondria pyruvate carrier (MPC) inhibitor.

Reportedly, mitochondria biogenesis and mitochondria membrane potential (MMP) are indispensable for hRPE differentiation^22–25^. In addition, a loss of mitochondrial mass and intact internal structures, as well as a decreased content of proteins involved in the electron transport chain (ETC), has been reported in hRPE cells obtained from AMD donor eyes^26–28^.

In early-stage AMD, RPE cells constitute cellular heterogeneity with dysfunctional and degenerated RPE cells^29–31^. Maintaining the dominancy of RPE cell subpopulations highly positive for MMP is critical, considering the perturbation of hRPE cell homeostasis by decreased cellular MMP^32,33^. Inhibition of ETC by complex I inhibitors, such as rotenone, has been shown to impair mitochondrial functions and affect the cell fate for differentiation^34–37^, although nothing has been known neither for the RPE cell differentiation nor for the effect of ETC inhibition on the composition of RPE cell subpopulations with distinct differentiation (or degeneration) status.

It is also speculated that hRPE tissue in AMD cases is composed of a single layer of heterogeneous cells at various levels of differentiation disposition or degeneration depending on the degree of mitochondrial damage^38–40^, and that the integrity of the tissue is influenced by external paracrine factors from neighboring cells^41^. The study on how localized RPE cell foci with differentiated phenotypes spread throughout the overall RPE tissue layer may contribute to understanding how RPE cells interact with each other as monolayer cells.

In this study, we investigated how the differentiation of individual cells involved in RPE cell clusters is regulated through up- and down-regulation of MMP, by comparing the effects of NAM (a stimulator of mitochondrial metabolism) and rotenone (a mitochondrial ETC inhibitor).

## Materials and methods

### Cell culture

Dulbecco’s Modified Eagle Medium/Ham’s F12 nutrient mixture (DMEM/F12) with Gibco GlutaMAX Supplement, minimum essential medium alpha (MEM), fetal bovine serum (FBS), nonessential amino acids (NEAA), TrypLE Express Enzyme (1×), and penicillin-streptomycin (Pe-St) were obtained from Thermo Fisher Scientific (Waltham, MA, USA). N1 medium supplement, taurine, hydrocortisone, triiodothyronine, NAM, and rotenone were obtained from Sigma-Aldrich, Inc. (St. Louis, MO, USA). ARPE-19 cells^42^ were obtained from American Type Culture Collection, Manassas, VA, USA (CRL-2302, passage 21), and cultured on T75 flasks or 6-well tissue culture plates. ARPE-19 cells were thawed and cultured in DMEM/F12, 10% FBS, and 1% Pe-St. For medium comparison studies, the cells were passaged and cultured in a 6-well tissue culture plate (seeding density, 200,000 cells/2.0 ml/well) in one of the following three media: (1) standard medium: DMEM/F12 (DMEM/F12, 1% FBS, and 1% Pe-St); (2) differentiation medium: MEM-Nic (MEM with GlutaMAX Supplement [Thermo Fisher Scientific], 1% FBS, 1% Pe-St, 1% N1 supplement, taurine (0.25 mg/ ml), hydrocortisone (20 ng/ml), triiodothyronine (0.013 ng/ml), and 10 mM NAM)^17^; (3) MEM-Nic + rotenone (10 or 20 nM). The cells were maintained at 37 °C, 5% CO_2_, and the media were replaced with fresh media once per week.

### Measurement of the area and the number of cell clusters

Based on phase contrast images of ARPE-19 cells cultured in each medium (i.e., DMEM/F12 + DMSO, MEM-Nic + DMSO, or MEM-Nic + rotenone [10 or 20 nM]) on day 0, 7, 14, 21, and 28, the area of cell clusters and the number of cell clusters in one field of view (1.4 × 1.0 mm) were measured (*n* = 4 each) using ImageJ (National Institutes of Health, Bethesda, MD, USA) software. Cell clusters were defined as small and uniform cell size cell populations with a large nucleus/cytoplasm (N/C) ratio compared to the surrounding cells.

### Immunohistochemistry and western blotting

In this study, the primary antibodies used for immunocytochemistry included rabbit anti-neural-cadherin (N-cadherin) (sc-7939; Santa Cruz Biotechnology, Inc., Dallas, TX, USA), mouse anti-cellular retinaldehyde–binding protein (CRALBP) (MA1-813; Thermo Fisher Scientific), mouse anti-Bestrophin/BEST1 antibody (E6-6) (ab2182; Abcam Ltd., Cambridge, UK), and anti-Phalloidin (Alexa-Fluor 546 Phalloidin A22283; Invitrogen, Carlsbad, CA, USA). The secondary antibodies used were goat anti-mouse and goat anti-rabbit IgG conjugated to Alexa Fluor 594 (Invitrogen). For western blot analysis, the antibodies included rabbit anti-silent information regulator 1 (SIRT1) (SirT1 [D1D7] Rabbit mAb #9475; Cell Signaling Technology, Inc., Danvers, MA, USA), rabbit anti-SIRT3 (ab217319; Abcam), mouse anti-peroxisome proliferator-activated receptor-gamma coactivator-1α (PGC-1α) (PA5-72948; Invitrogen, Waltham, MA, USA), rabbit anti-mitochondrial transcription factor A (TFAM) (8076; Cell Signaling Technology), rabbit anti-translocate of outer mitochondrial membrane 20 (TOMM20) (NBP1-81556; Novus Biologicals, LLC, Centennial, CO, USA), mouse anti-COX IV (ab202554; Abcam) and anti-GAPDH (M171-3, MBL, Tokyo, Japan) (*n* = 3 each).

### Enzyme-linked immunosorbent assay (ELISA) for vascular endothelial growth factor (VEGF)

ARPE-19 cells were seeded in 12-well Transwell Cell Culture Plates (3513; Corning Inc., Corning, NY, USA) in DMEM/F12 or MEM-Nic, with the media being replaced with fresh media once per week. After 28 days, the supernatants in the inner inserts or outer chambers were collected (*n* = 3 each). VEGF protein levels were quantified using the DuoSet ELISA Development System (R&D Systems, Inc., Minneapolis, MN, USA) according to the manufacturer’s instructions.

### Lactate and adenosine triphosphate (ATP) assay

After 4 weeks of incubation in DMEM/F12 or MEM-Nic medium, and at 6 h prior to measurements being taken, the medium was replaced with that containing 2-deoxy-D-glucose (2DG) (Merck KGaA, Darmstadt, Germany) or dimethyl sulfoxide (DMSO). The L-Lactate Assay Kit (ab65330; Abcam) and ATP Assay Kit-Luminescence (A550; Dojindo Laboratories, Kumamoto, Japan) were used to measure total lactate and ATP levels in DMEM/F12 + DMSO, DMEM/F12 + 2DG, MEM-Nic + DMSO, and MEM-Nic + 2DG. ARPE-19 cells were seeded (10,000 cells/100 ml/well) in a 96-well culture plate (3595; Corning) according to the manufacturer’s instructions (*n* = 3 each). The optical density in the lactate was assessed using a Thermo Scientific Multiskan SkyHigh Spectrophotometer (Thermo Fisher Scientific) at a wavelength of 570 nm, and luminescence in ATP was measured using a GloMax Explorer Multimode Microplate Reader (Promega Corp., Madison, WI, USA).

### Assessment of mitochondrial respiratory capacity

For the assessment of mitochondrial respiratory capacity, ARPE-19 cells were seeded (80,000 cells/100 ml/well) in a Seahorse XFe24 Analyzer (Agilent Technologies, Inc., Santa Clara, CA, USA) with a 24-well plate. The cells then underwent the XFe24-analyzer ‘Cell Mito Stress Test’ according to the manufacturer’s protocol (*n* = 4 each). The XF assay media was supplemented with 10 mM glucose, 1 mM sodium pyruvate, and 2 mM L-glutamine. ETC inhibitors were sequentially added to modulate oxygen consumption for mitochondrial function assessment; oligomycin (1 µM), Carbonyl cyanide-4-(trifluoromethoxy) phenylhydrazone (FCCP) (1 µM), and rotenone + antimycin A (both, 0.5 µM).

### Measurement of MMP

Changes in MMP were detected using the JC-1 MitoMP Detection Kit (Dojindo Laboratories) as previously described. Briefly, after washing the cells with Hanks’ balanced salt solution, they were analyzed using the BD FACSCanto II Clinical Flow Cytometry System (BD Biosciences, Franklin Lakes, NJ, USA). For fluorescence imaging analysis, the cells were first incubated with 2 mM JC-1 (a cationic carbocyanine fluorescent dye when added to living cells) for 30 min at 37 °C, and then analyzed using a fluorescence microscope system (BZ X-700; Keyence Corp., Osaka, Japan), with red stains indicating JC-1 aggregates (dimers) in intact mitochondria and green stains indicating JC-1 monomers in apoptotic cells with the depolarization of MMP. JC-1 fluorophores accumulate in mitochondria in a potential-dependent manner, shifting from green (488 nm) to red (561 nm), and mitochondrial hyperpolarization is indicated by an increase in the red: green fluorescence intensity ratio. For medium comparison studies, the cells were cultured in a 6-well tissue culture plate (seeding density, 200,000 cells/well) in one of the following media: (1) DMEM/F12 + DMSO, (2) MEM-Nic + DMSO, and (3) MEM-Nic + rotenone (10 or 20 nM).

### Statistical analysis

The data are presented as mean ± SD. All studies were performed using at least three sets of independent experiments. For all experiments, the numbers of samples used are specified in the respective figure legends. The distribution of the data was examined using the Shapiro–Wilk normality test. A statistical analysis of differences was performed using Student’s *t*-test for comparisons between two groups. For analysis of variance, this was followed by either Tukey’s or Dunnett’s test. The values shown in the graphs represent the mean ± SD. In all cases, a *P*-value of < 0.05 was considered statistically significant.

## Results

### NAM-containing medium expanded cell clusters with smaller RPE cells

**Fig. 1:**
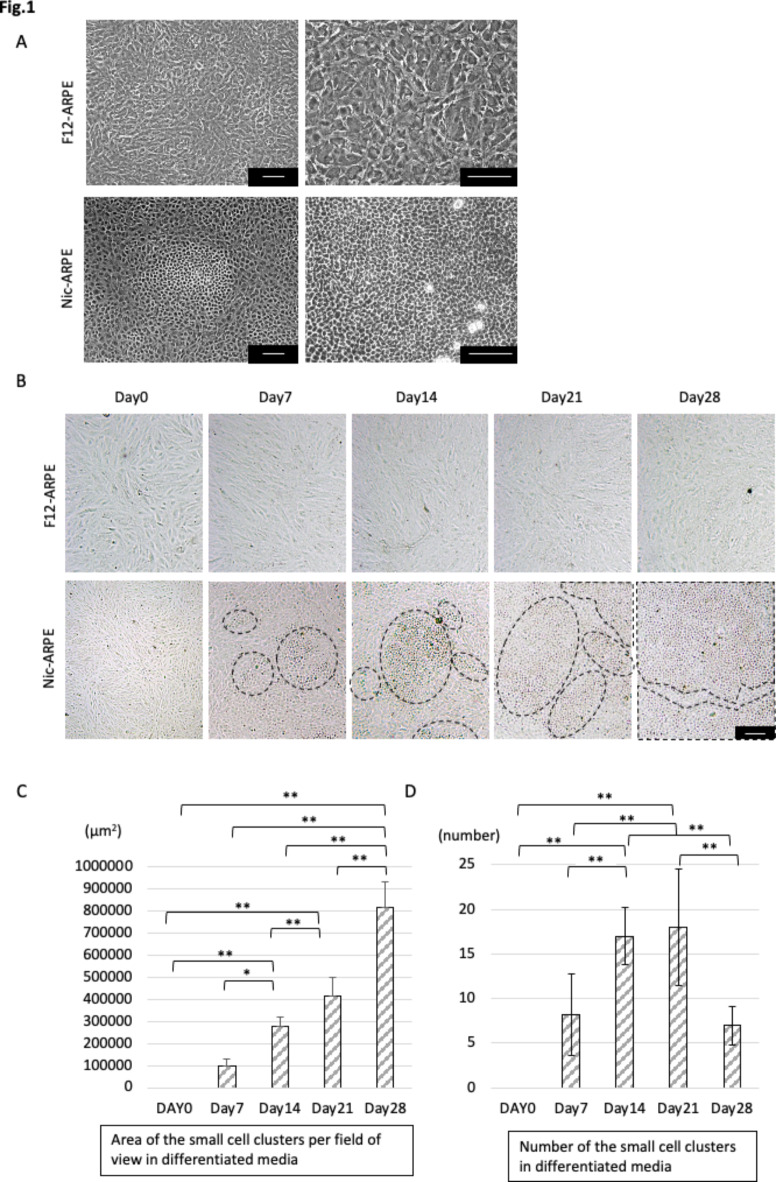
Changes in cell morphology and cell clusters in differentiation medium. (**A**) Phase contrast microscopy images of ARPE-19 cell-line cells incubated for 14 days in Dulbecco’s Modified Eagle Medium/Ham’s F12 nutrient mixture (DMEM/F12) medium (top row) or in Minimum Essential Medium Alpha (MEM) with nicotinamide (NAM) (MEM-Nic) medium (bottom row). Scale bar: 100 μm. (**B**) Images showing the change in the morphology of the ARPE-19 cells cultured for 4 weeks in DMEM/F12 (top row) and MEM-Nic (bottom row). The clusters composed of smaller cells are outlined by a dashed line. Scale bar: 100 μm. (**C**,** D**) The area of cell clusters and numbers of clusters in one field of view (1.4 × 1.0 mm) were compared in each time period. The area occupied by the smaller cell clusters in the Nic-ARPE cells increased over the culture period, and the number of the clusters composed of smaller cells in the Nic-ARPE cells peaked at between 14 and 21 days, and then declined (*P < 0.05, **P < 0.01, *n* = 4).

Cell morphology was monitored periodically by culturing ARPE-19 cells in standard medium (DMEM/F12) or differentiation medium (MEM-Nic) for 4 weeks as previously reported^17,21^. Gross morphological examination of the cells cultured on plastic surfaces revealed the presence of heterogeneous cells in the context of morphology. ARPE-19 cells cultured in standard medium (hereinafter termed F12-ARPE cells) showed a fibroblast-like morphology throughout the culture plates. On the other hand, ARPE-19 cells cultured in the presence of differentiation medium (hereinafter termed Nic-ARPE cells) revealed the presence of cells smaller in size than the F12-ARPE cells throughout the culture plates and formed in hexagonal lattices, with far smaller cells in the clusters (Fig. 1A). In addition, pigmentation of cells in clusters was observed (Fig. 1B). When ARPE-19 cells were cultured for 4 weeks, clusters composed mostly of smaller size cells were detected in the Nic-ARPE cells after 1 week and the area occupied by those clusters increased significantly over the culture period. However, they were not observed in the F12-ARPE cells (Fig. 1B,C). The number of clusters composed of smaller size cells in the Nic-ARPE cells peaked at between 2 and 3 weeks, and then declined (Fig. 1D), thus suggesting that the increase in the number of clusters observed over the culture period might be followed by the fusion with neighboring clusters to generate a larger area of clusters (see also Fig. 1B). Next, we focused on the heterogeneity of the differentiation of RPE cells in and outside of those cell clusters.

### Small cell clusters expressed RPE cell differentiation markers

**Fig. 2:**
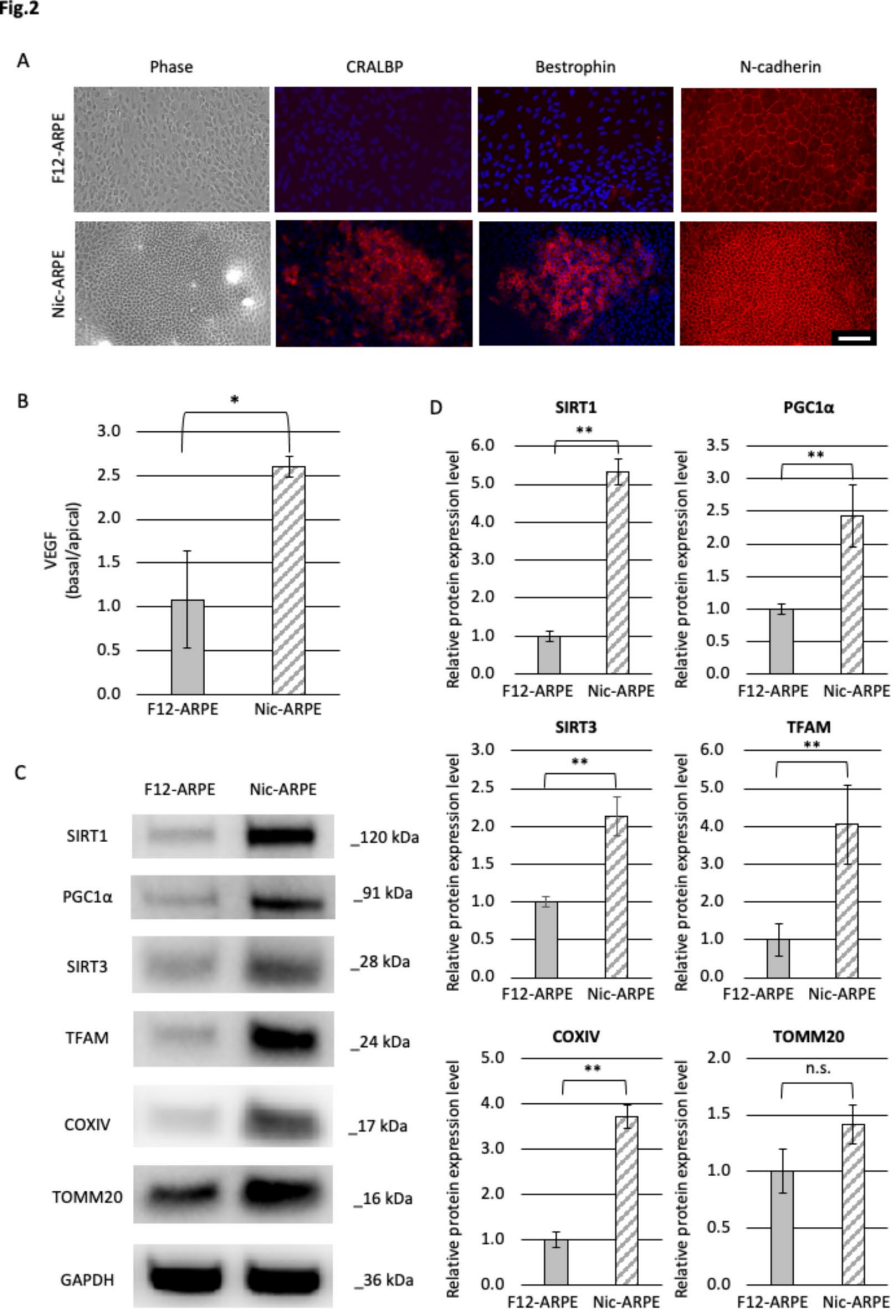
Retinal pigment epithelium cell differentiation and mitochondria-related protein. (**A**) Phase contrast images and immunofluorescence micrographs of cellular retinaldehyde–binding protein (CRALBP), Bestrophin and N-cadherin labeling in ARPE-19 cells incubated for 28 days in DMEM/F12 (top row) and MEM-Nic (bottom row). Scale bar: 100 μm. (**B**) Vascular endothelial growth factor secretion to apical or basolateral side was measured using cultures in Corning Transwell cell culture plates. Cultures in MEM-Nic showed the polarized secretion to the basolateral side (* *P* = 0.04, *n* = 3). (**C**) Protein expression levels of SIRT1, PGC-1α, SIRT3, TFAM, COX IV, and TOMM20 in ARPE-19 cells incubated for 28 days in standard medium (DMEM/F12) or differentiation medium (MEM-Nic) were determined by western blotting. Molecular mass in kDa is indicated at the right of the quantification of the blots. (**D**) Quantification of blots in (**C**) are shown in (**D**). Expressions of all markers other than TOMM20 were more elevated in gross cells cultured in MEM-Nic than in DMEM/F12 (**P < 0.01, *n* = 3).

To confirm the gross expression of ARPE-19 cell differentiation markers, immunohistochemical examination was used to investigate the protein extracts from gross cultured ARPE19 cells, and found RPE cell differentiation markers CRALBP and Bestrophin were more highly expressed in the smaller size cells in cluster of Nic-ARPE cells, but not in F12-ARPE cells. N-cadherin was also expressed in both cell groups (Fig. 2A). Next, to clarify whether the polarization of the cells would be accompanied during the cultures, polarized production of VEGF secretion either to the basolateral or apical side was addressed. The mean ratio of VEGF secretion to the basal to apical side in the F12-ARPE cells and Nic-ARPE cells was 1.08 ± 0.56 and 2.59 ± 0.12, respectively (*P* = 0.04, *n* = 3), thus clearly indicating higher cell polarity in the Nic-ARPE cells compared to the F12-ARPE cells (Fig. 2B). Based on the comparison of the basal and apical secretion ratios of VEGF between the Nic-ARPE cells and the F12-ARPE cell populations, the results suggest that the Nic-ARPE cells may have a higher degree of disposition to differentiate.

### Mitochondria-related proteins were increased in differentiated RPE cells

The expression of mitochondria-related proteins was examined using Western blotting. SIRT1, PGC-1α, SIRT3, TFAM, and COX IV were elevated in the gross Nic-ARPE cells than in the F12-ARPE cells (*P* < 0.0001, *P* = 0.0072, *P* = 0.0019, *P* = 0.0093, and *P* = 0.0001, respectively), and no significant difference was observed in TOMM20 (*P* = 0.0516) (Fig. 2C,D). This indicates that mitochondrial biogenesis, transcription factors, and respiratory capacity were enhanced in differentiated RPE cells.

### Mitochondrial respiration in differentiated RPE cells

**Fig. 3:**
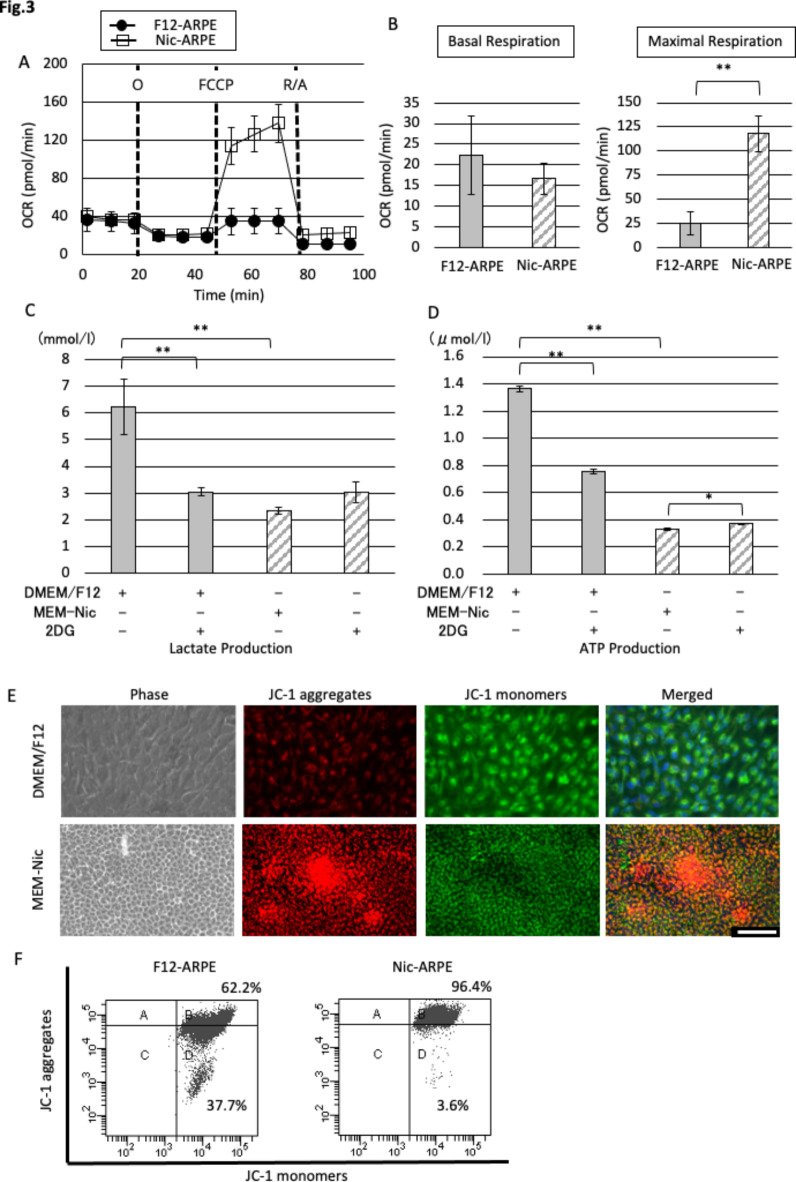
Mitochondrial respiration and mitochondrial membrane potential (MMP). (**A**) Comparison of mitochondria respiration between ARPE-19 cells incubated for 28 days in DMEM/F12 and in MEM-Nic. Dashed lines indicate the injection time point of inhibitory reagents; O, Oligomycin; FCCP, Carbonyl cyanide-4-(trifluoromethoxy) phenylhydrazone; R/A, Rotenone/Antimycin A; OCR, oxygen consumption rate. (**B**) Graphs showing basal and maximal respiration (**P < 0.01, *n* = 4). (**C**,**D**) Lactate and adenosine triphosphate production were measured in the cultures in DMEM/F12 or MEM-Nic in the presence or absence of 0.5 mM 2-Deoxy-D-glucose (2DG), which selectively blocks the glycolytic system (*P < 0.05, **P < 0.01, *n* = 3). (**E**) Mitochondria of ARPE-19 cells cultured in the same media as used for A were stained with JC-1 dye. Scale bar: 100 μm. Red staining indicates JC-1 aggregates (dimers) in intact mitochondria, and green staining indicates JC-1 monomers in apoptotic cells with the depolarization of MMP. JC-1 fluorophores accumulated in mitochondria in a potential-dependent manner, shifting from green (488 nm) to red (561 nm). (**F**) MMP of ARPE-19 cells cultured in DMEM/F12 or MEM-Nic were compared by flow cytometry.

Mitochondrial respiratory capacity was also analyzed for the gross cultured ARPE-19 cells. In the F12-ARPE and Nic-ARPE cells, the mean basal oxygen consumption rates (OCR) were 22.3 ± 9.5 and 16.7 ± 3.8 pmol/minute, respectively, thus illustrating no statistically significant difference in OCR between the F12-ARPE and Nic-ARPE cells. However, the maximal OCR were 24.8 ± 12.4 and 117.8 ± 18.9 pmol/minute, respectively, i.e., significantly higher in the Nic-ARPE cells (*P* = 0.0005) (Fig. 3A,B). The higher aerobic respiration capacity and oxygen demand in the Nic-ARPE cells suggests that the energy metabolism of those cells is dominant in aerobic respiration.

It has been reported that energy metabolism in normal hRPE cells is mainly aerobic, whereas primary hRPE cells from AMD donors are inclined towards glycolysis^43,44^. In this study, 2DG was used to block the glycolytic pathway. In DMEM/F12, DMEM/F12 + 2DG, MEM-Nic, and MEM-Nic + 2DG, the mean ± SD lactate produced was 6.23 ± 1.04, 3.03 ± 0.15, 2.35 ± 0.14, and 3.04 ± 0.40 mmol/L, respectively, and the mean ± SD ATP produced was 1.36 ± 0.02, 0.75 ± 0.02, 0.32 ± 0.01, and 0.37 ± 0.01 µmol/L, respectively (Fig. 3C,D). Significantly more lactate and ATP was produced in the F12-ARPE cells than in the Nic-ARPE cells (*P* < 0.0001). The morphology of 4 cultured cells (i.e., on day 28) is shown in Supplementary Fig. S3. 2DG significantly decreased the production of both lactate and ATP in the F12-ARPE cells (lactate: *P* = 0.0005, ATP: *P* < 0.0001), but not in the Nic-ARPE cells, and increased ATP production in the Nic-ARPE cells (*P* = 0.026) (Fig. 3C,D). These findings indicate that the respiration of the ARPE cells was reprogrammed mostly to aerobic respiration rather than glycolysis by NAM-containing differentiation medium, consistent with the findings previously reported^21^, although the culture methods used in their study were different and far more complicated.

### Mitochondria membrane potential (MMP) is enhanced in differentiated smaller RPE cells

Our immunohistochemical findings revealed that CRALBP and Bestrophin were expressed only in smaller cells in the cluster of Nic-ARPE cells (Fig. 2A). The localization of cells with high MMP was similarly investigated by JC-1 staining. Compared with the F12-ARPE cells, the Nic-ARPE cells contained cells with increased red JC-1 fluorescence, especially in the clusters (Fig. 3E). These findings indicate that RPE cell differentiation indexed by CRALBP, Bestrophin, and MMP was accelerated in the smaller RPE cells in the clusters generated in NAM containing differentiation medium, suggesting the presence of a higher proportion of differentiated RPE cells with functional mitochondria.

Flow cytometry was used to reveal the heterogeneity of Nic-ARPE cells in terms of MMP adapted for mitochondrial Flux analysis (Fig. 3A,B). The cutoff value for cells indicating high MMP was set based on the data obtained by adding FCCP (Supplementary Fig. S1). The proportion of F12-ARPE cells with high MMP was 62.2%, whereas the proportion of Nic-ARPE cells with high MMP was 96.4% (Fig. 3F and Supplementary Fig. S2), thus suggesting that NAM increased functional mitochondria. MMP distinguished by JC-1 staining revealed that Nic-ARPE cells are relatively homogeneous in the expression of functional mitochondria compared to F12-ARPE cells (Fig. 3E,F).

### Respiratory chain complex I inhibitor decreased MMP and inhibited ARPE-19 cell differentiation

**Fig. 4:**
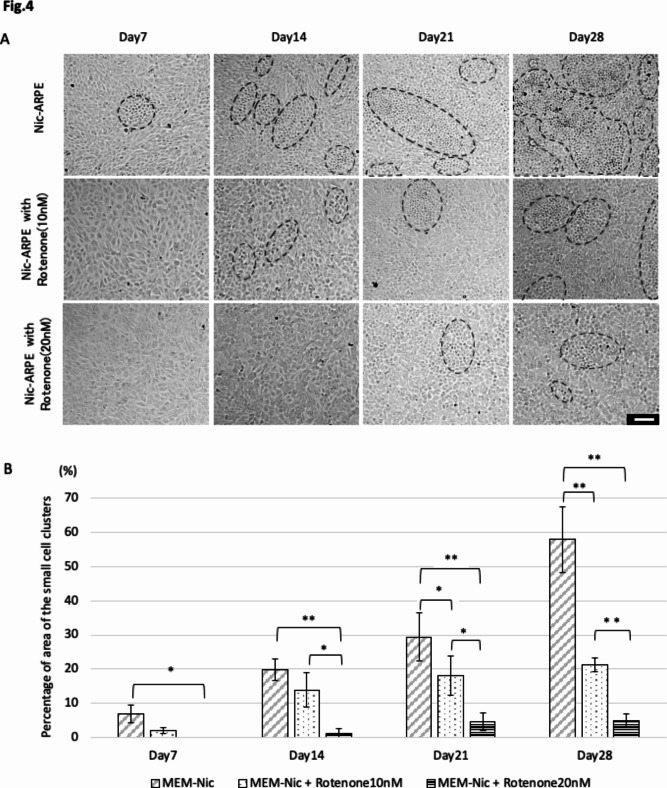
Inhibition of cell cluster generation by mitochondria complex 1 inhibitor. (**A**) Phase contrast images of ARPE-19 cells incubated for 7, 14, 21, and 28 days in MEM-Nic with or without rotenone, which inhibits electron transfer chain complex I in the mitochondrial inner membrane. Scale bar: 100 μm. (**B**) The ratio of the area of cell clusters composed of smaller cells in one field of view (1.4 × 1.0 mm) in the culture in the presence of rotenone was greatly reduced in a dose-dependent manner (*P < 0.05, **P < 0.01, *n* = 4).

Since respiratory chain complex I in the mitochondrial inner membrane is important for MMP formation, we investigated the effects of mitochondria complex I on MMP and RPE cell differentiation using rotenone, a widely approved mitochondrial ETC complex I inhibitor. Briefly, we first examined the concentration at which rotenone causes ARPE-19 cell death, and found significant cell death at a concentration of 100 nM or more, whereas no cell death occurred at concentrations below 20 nM (Supplementary Fig. S4). Next, ARPE-19 cells were cultured for 4 weeks in NAM-containing differentiation medium in the presence of rotenone (0, 10, and 20 nM). In cells cultured in medium without rotenone, the area of the cell clusters increased over the culture period, while the addition of rotenone decreased the ratio in a concentration-dependent manner; the ratio of the area of the cell clusters with rotenone (0, 10, and 20 nM) were 7.0 ± 2.7, 2.1 ± 0.8 (*P* = 0.13), 0.0 ± 0.0% (*P* = 0.033, 0.51) on day 7, 19.9 ± 3.1, 13.9 ± 5.1 (*P* = 0.064), 1.1 ± 1.6%(*P* < 0.001, *P* = 0.011) on day 14, 29.3 ± 7.0, 18.1 ± 5.8 (*P* = 0.040), 4.6 ± 2.6% (*P* < 0.001, *P* = 0.006) on day 21, 58.0 ± 9.6, 21.3 ± 1.9 (*P* < 0.001), 4.9 ± 2.1% (*P* < 0.001, *P* < 0.001) on day 28. In fact, cell clusters were rarely observed in the cells cultured in medium supplemented with 20 nM rotenone (Fig. 4A,B, and Supplementary Fig. S5).

**Fig. 5:**
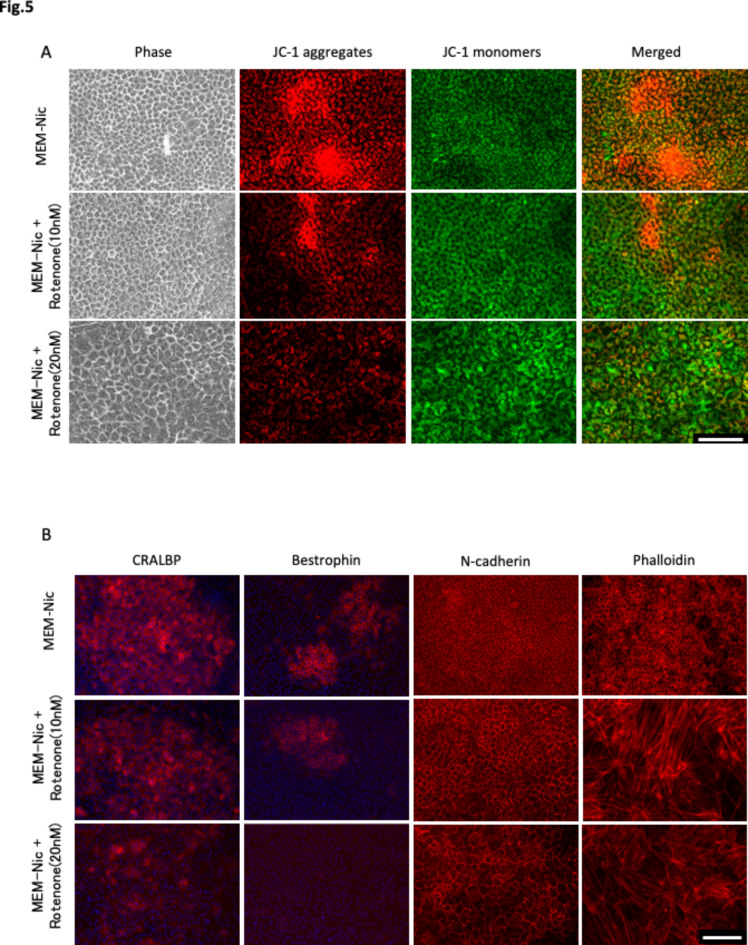
Rotenone inhibits ARPE-19 cell differentiation. (**A**) Mitochondria of ARPE-19 cultured in MEM-Nic, MEM-Nic + rotenone (10 nM), or MEM-Nic + rotenone (20 nM) was stained with JC-1 to investigate the changes of MMP as explained in Fig. 3E. (**B**) Immunofluorescence micrographs of CRALBP, Bestrophin, N-cadherin and Phalloidin labeling in ARPE-19 cells incubated for 28 days in the same media as used for (**A**). Scale bar: 100 μm.

Next, we examined the effect of rotenone on MMP and ARPE-19 cell differentiation. ARPE-19 cells were cultured in NAM-containing differentiation medium with or without rotenone for 28 days. As described above, increased red fluorescence of JC-1, expression of CRALBP and Bestrophin and lateral circumferential expression of F-actin of the cell were observed in the clusters in medium without rotenone, whereas the JC-1 red and differentiation marker fluorescence were decreased in a concentration dependent manner and a diverse F-actin arrangement were observed in medium with rotenone (Fig. 5A,B, and Supplementary Fig. S6).

## Discussion

This study revealed that cultured ARPE-19 cells generated cell clusters composed mostly of differentiated smaller size cells only in the NAM-containing differentiation medium. The enhanced expression of MMP was detected only in those clusters (Fig. 3E). The area of the clusters expanded from around 7–58% of the culture dish surfaces during the 1- to 4-week culture period in the presence of NAM, compared to null % in the absence (Fig. 1C). The numbers of clusters peaked at 2 weeks after the initiation of the cultures (Fig. 1D), indicating the expansion of the cluster areas by fusion among preformed clusters. Rotenone, a proven ETC complex I inhibitor, disrupted the ordered ARPE-19 cell morphology with lateral circumferential F-actin (Fig. 5B) and inhibited the generation of those cell clusters (Fig. 4A,B), accompanied by repressed MMP (Fig. 5A).

Normally, hRPE tissue is a monolayer of cells closely linked to the photoreceptors. During retinal detachment resulting from a loss of normal matrix adhesion and changes in the cell-cell adhesion profile, RPE cells become dislodged from the monolayer and undergo EMT to a fibrotic phenotype^29–31^. The generation of cell clusters composed mostly of differentiated and/or differentiation disposed cells with RPE cell markers in the presence of NAM (Fig. 2A) may implicate the protection of differentiated ARPE-19 cells by maintaining the normal matrix adhesion or cell-cell contact to maintain the close cell to cell interplays (Fig. 1B,C). The area of cell clusters composed of smaller cells was found to spread spatially over a culture period in the presence of NAM (Fig. 1B,D). We reported the presence of paracellular interplay to induce spreading of the degenerated hRPE subpopulations from the degenerated foci into neighbors in single-layer RPE tissues^20^. This paracellular interplay may be prevented by extracellular vesicle microRNA (miR). It has been reported that in single-cell-layer corneal endothelial tissue, the paracellular interplay through miR-184 is implicated^45^; for convenience, that observation is detailed in Supplementary Information 2. Moreover, Jiang et al.^41^ described that, albeit partly, miR-184 is involved in promoting RPE differentiation in the pathogenesis of dry AMD.

In our previous study, we reported that the mitochondrial functional phenotypes of hRPE cells are plastic and implicate the presence of fragile differentiated phenotypes that are reminiscent of the presence of subpopulations in in vivo hRPE tissues from healthy to an aging or diseased state, such as AMD^20^. Converging evidence implicates mitochondrial dysfunctions in the pathogenesis of AMD^26–28^. In early-stage AMD, hRPE cells constitute cellular heterogeneity with degenerated and non-degenerated RPE cells. Mitochondria are important organelles playing relevant roles in cell development and differentiation^46,47^. Consistent with the previous findings^17,21^, the presence of NAM in cultures induced higher expression of RPE differentiation markers and proteins involved in mitochondrial biogenesis, and enhanced mitochondrial respiratory capacity, dependent on aerobic respiration (Figs. 2A,C,D and 3A,B).

Dysfunctional mitochondria induce increased levels of reactive oxygen species (ROS), mitochondrial DNA damage, and defective metabolic activity^39,48,49^. Inhibition of mitochondria ETC complex I by an inhibitor such as rotenone is accompanied by ROS generation, leading to deterioration of cell morphology and loss of function. Low phagocytic RPE cells have been reported to lack circumferential F-actin, which is present in high phagocytic RPE cells^50^. In our study, the disappearance of circumferential F-actin upon addition of rotenone, which was present with Nic-ARPE-19 cells, suggests that ETC complex I is involved in RPE differentiation.

A major role in mitochondrial biogenesis and oxidative metabolism is played by PGC-1α^51^. Silent information regulator T1 (SIRT1) belongs to a family of class II histone/protein deacetylase proteins, and is the only protein able to deacetylate and activate PGC-1α^52^. A repressed SIRT1/PGC-1α pathway and mitochondrial disintegration plays a critical role in the pathogenesis of AMD^48^. Considering the lower expression levels of PGC-1α/SIRT1 in the diseased AMD cells^48^, the resilience of the SIRT1/PGC-1α pathway in Nic-ARPE-19 cells, as well as the polarized basolateral secretion of VEGF (Fig. 2B,C), may support the notion that the smaller cells in the cell clusters here designated would be functionally integral hRPE cells. However, to clarify this concept, it is necessary to more accurately measure the size of cells and confirm the involvement of these cells in differentiation. In addition, as reported by Hazim et al.^17^ and Dunn et al.^42^, further investigation of the polarity of these small cells through VEGF immunostaining and apical FGF5 secretion experiments is required.

Alterations on mitochondria physiology imply the maintenance of the import of carbon sources to sustain the tricarboxylic acid (TCA) cycle, ETC, and ultimately ATP synthesis. However, the relative participation of TCA metabolites and/or protons produced in ETC has yet to fully elucidated. Intracellular production of ATP and lactate using 2DG indicated that the addition of NAM polarized energy metabolism from glycolysis-dependent to oxidative phosphorylation-mediated metabolism (Fig. 3C,D). Glycolysis leads to production of pyruvate, which can be transported into the mitochondria via an MPC.

In this current study, we used rotenone, a mitochondrial ETC complex 1 inhibitor that affects mitochondrial function, to demonstrate that MMP is crucial for the differentiation of ARPE-19 cells. On the other hand, the use of the MPC inhibitor UK5099 can allow for the study of different aspects of mitochondrial function. In fact, Hazim et al.^17,21^ recently suggested a link between the mitochondrial metabolism of the ARPE-19 cells and its state of differentiation as a polarized epithelium, by using UK5099 and FK866. It should be kept in mind that the NAM phosphoribosyl-transferase inhibitor FK866 unexpectedly showed an increased consumption of pyruvate and succinate for energy production^53^. The blockage of MPC, and thus the uncoupling of glycolysis and mitochondrial respiration, may result in higher pyruvate accumulation in cytoplasm, thereby making it possible to keep nicotinamide adenine dinucleotide^+^ levels high in the cytoplasm through the action of lactate dehydrogenase A subunit^54^. Blocking MPC would prevent not only the import of pyruvate but also of the proton, which favor the maintenance of a higher MMP and ultimately ATP production by mitochondria complex V.

To validate the concept proposed in this study that ARPE-19 cells with high MMP express RPE cell differentiation markers with small cell size localized in cell clusters would be certainly disposed to differentiated mature RPE cells, further research is needed to clarify the correlation of the ARPE-19 cell differentiation with the more quantitative cell size measurements in and outside cell clusters in cultures. Moreover, additional studies are needed to more clearly elucidate and better understand the roles of mitochondrial function and ROS in this context.

## Electronic supplementary material

Below is the link to the electronic supplementary material.

Supplementary Material 1

## Data Availability

The datasets generated during the current study are available from the corresponding author on reasonable request.
