# Supplementary material for: Clustered ARPE-19 cells distinct in mitochondrial membrane potential may play a pivotal role in cell differentiation
Source: Sci Rep. 2024 Sep 27;14:22391. doi: 10.1038/s41598-024-73145-w (PMC11436949; doi:10.1038/s41598-024-73145-w)

Supplementary Information

Clustered ARPE-19 cells distinct in mitochondrial membrane potential may play a pivotal role in cell differentiation

Takafumi Miyatani[1], Hiroshi Tanaka[1*], Kosaku Numa[1], Asako Uehara[1], Yohei Otsuki[1], Junji Hamuro[1], Shigeru Kinoshita[2] & Chie Sotozono[1]

[1]Department of Ophthalmology and [2]Department of Frontier Medical Science and Technology for Ophthalmology, Kyoto Prefectural University of Medicine, 465 Kajii-cho, Hirokoji-agaru, Kawaramachi-dori, Kamigyo-ku, Kyoto 602-0841, Japan

* Correspondence: Hiroshi Tanaka, MD, PhD, Department of Ophthalmology, Kyoto Prefectural University of Medicine, 465 Kajii-cho, Hirokoji-agaru, Kawaramachi-dori, Kamigyo-ku, Kyoto 602-0841, Japan.
Tel.: +81-75-251-5578, Fax: +81-75-251-5663, E-mail: htanakan@koto.kpu-m.ac.jp

Supplemental Figure 1

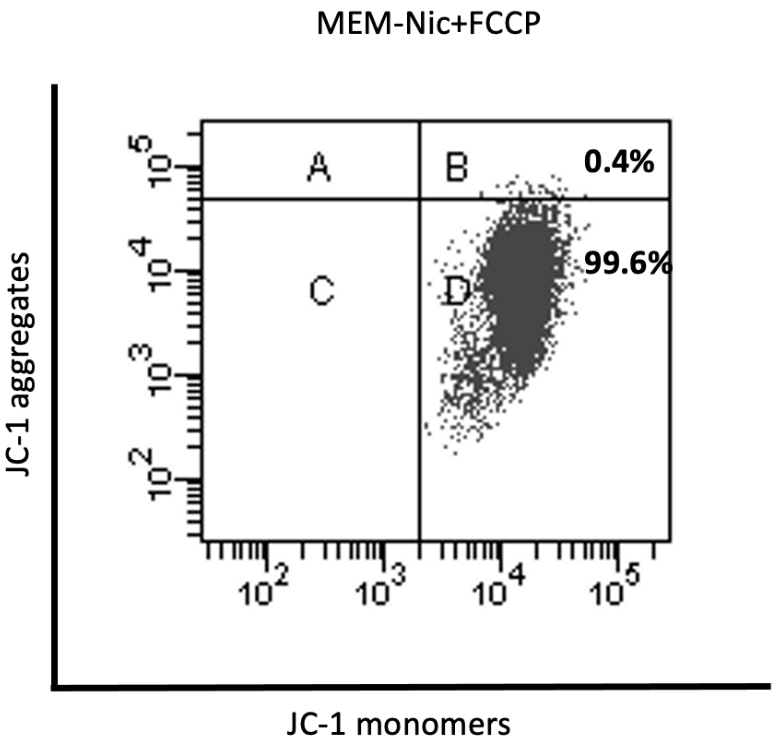

<u>**Supplemental Figure 1:**</u> Cutoff values for ARPE-19 cells in JC-1 aggregates in FACS. ARPE-19 cells were seeded into a 6-well plate in MEM-Nic with a cell density of 200,000 cells/well, and then cultured for 28 days. The collected cells were incubated with 2 mM JC-1 and 100 µM FCCP for 30 minutes at 37°C, and then analyzed using the BD Biosciences FACSCanto II Clinical Flow Cytometry System for fluorescence FITC (green) and PE (red). The uncoupling agent FCCP has been associated with mitochondrial inhibition and activates ionic currents and depolarizes the plasma membrane potential; 100 µM FCCP was added to RPE and MMP depolarized cells were used as negative controls.

Supplemental Figure 2

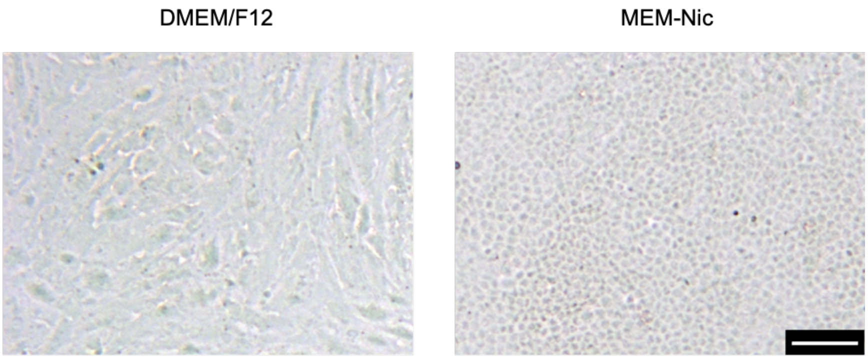

Supplemental Figure 2: Cell morphology prior to flow cytometry. Phase-contrast microscopy images of ARPE-19 cells cultured for 28 days in DMEM/F12 (left) and MEM-Nic (right) used in flow cytometry. ARPE-19 cells cultured in DMEM/F12 showed a fibroblast-like morphology throughout the culture plates, while ARPE-19 cells cultured in MEM-Nic showed the presence of smaller cells than those throughout the culture plates and formed hexagonal lattices, with far smaller cells in the clusters. Scale bar: 100 μm.

Supplemental Figure 3

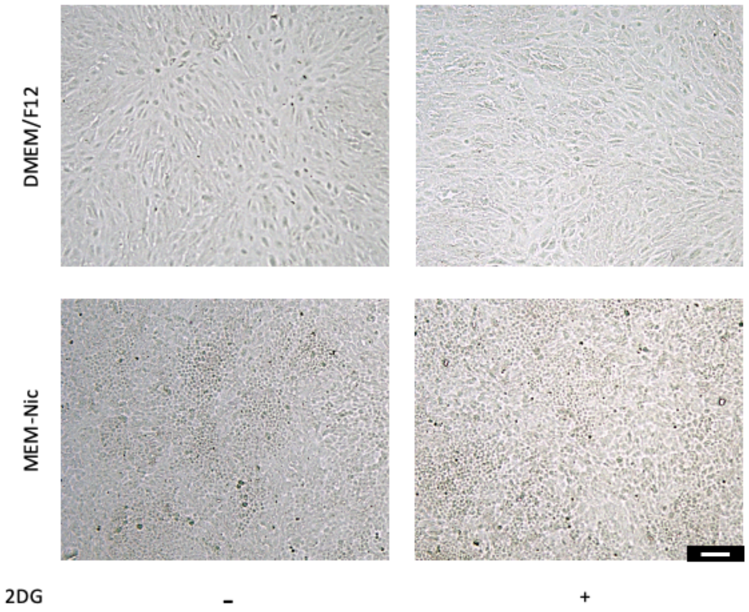

<u>Supplemental Figure 3:</u> Phase-contrast microscopy images of ARPE-19 cells used for the determination of lactate and ATP produced. Images showing the cell morphology of ARPE-19 cells after 28-day culture and the addition of 2DG for 6 hours in DMEM/F12 medium (top left) or MEM-Nic medium (bottom left). Addition of 2DG showed no apparent change in cell morphology in the cells of either culture medium. Scale bar: 100 µm.

Supplemental Figure 4

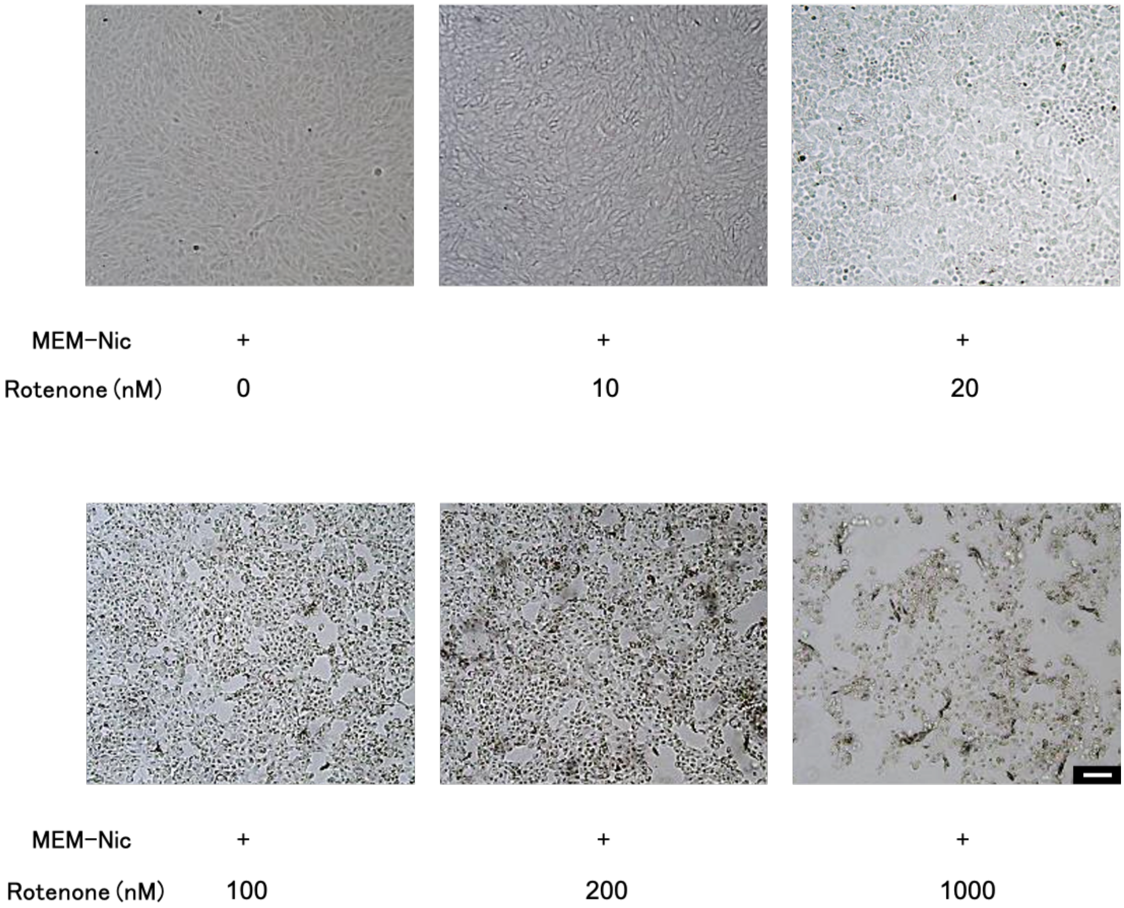

Supplemental Figure 4: Determination of the rotenone concentration that does not cause cell death. Phase-contrast microscopy images of cultured ARPE-19 cells at 2 days after the addition of rotenone (0 nM [top left], 10 nM [top middle], 20 nM [top right], 100 nM [bottom left], 200 nM [bottom middle], and 1000 nM [bottom right]). ARPE-19 cells were cultured for 28 days before the addition of rotenone in MEM-Nic. ARPE-19 cells cultured with rotenone above 100 nM showed cell death due to rotenone addition, but not below 20 nM. Scale bar: 100 µm.

Supplemental Figure 5

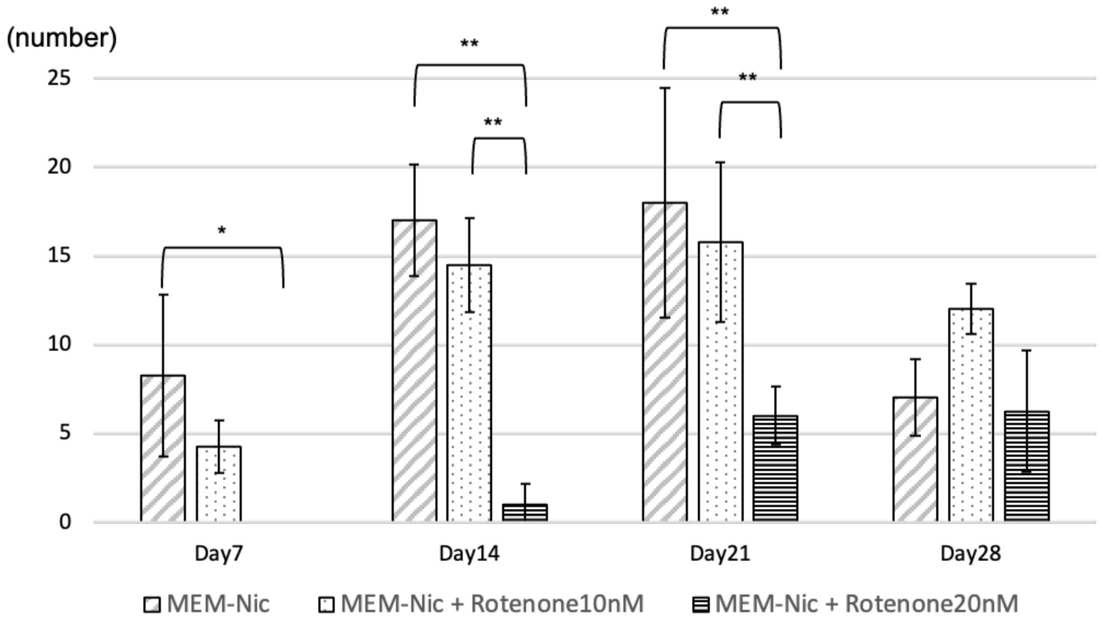

<u>**Supplemental Figure 5:**</u> Periodical changes in the number of clusters composed of smaller cells. The number of clusters composed of smaller cells in one view field (1.4 x 1.0 mm) cultured in MEM-Nic (rotenone addition; 0, 10, and 20 nM) was compared. The numbers of the clusters were significantly reduced progressively until day 21 when cultured in MEM-Nic + rotenone (20 nM) compared to those in (MEM-Nic or MEM-Nic + rotenone (10 nM). However, on day 28, no significant difference was detected (*: $P<0.05$, **: $P < 0.01$).

Supplemental Figure 6

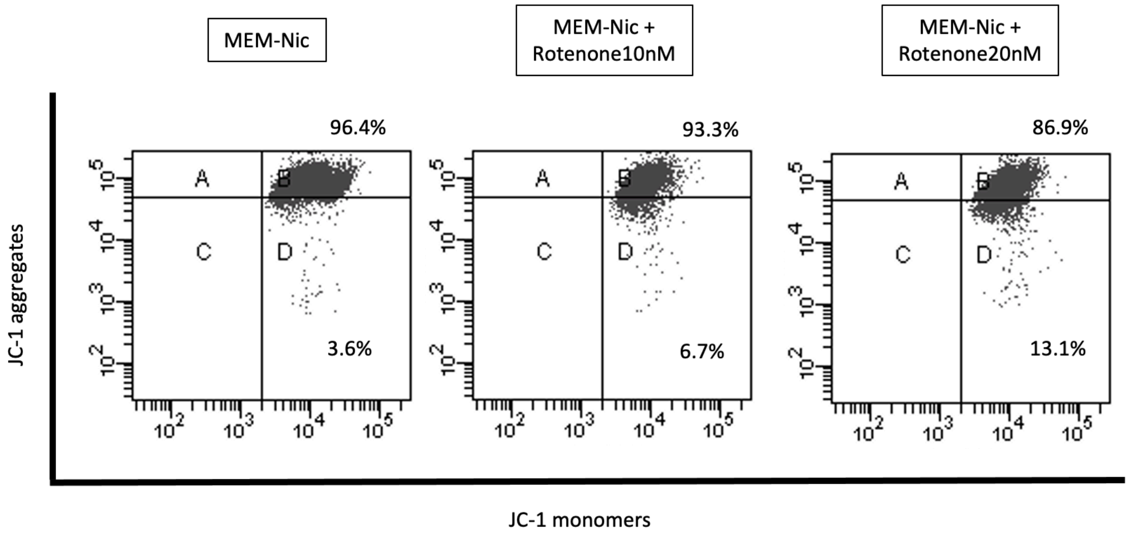

<u>Supplemental Figure 6:</u> Changes in MMP by rotenone. MMP of ARPE-19 cells cultured in MEM-Nic with or without rotenone were compared quantitatively by flow cytometry. The percentage of mitochondria with high MMP was 96.4% in MEM-Nic, 93.3% in MEM-Nic + rotenone (10 nM), and 86.9% in MEM-Nic + rotenone (20 nM).

Supplementary Information 1

It has been reported that RPE cells *in vivo* measure approximately 15-25 μm (Panda-Jonas, S. *et al*. Retinal pigment epithelial cell count, distribution, and correlations in normal human eyes. *Am. J. Ophthalmol*. **121**, 181-189 [1996]). In this current study, the small cells were approximately 10-20 μm in size, which is close to the size of cells *in vivo*. Our findings suggest that smaller cells do indeed exhibit higher MMPs and higher expression of CRALBP and Bestrophin, suggesting that cell size plays some role in differentiation.

Moreover, cell competition is an important mechanism whereby the cell society robustly orchestrates tissue homeostasis, through cell death, cellular senescence, or cell extrusion due to physical pressures. Therefore, human corneal endothelial cell (HCEC) subpopulations with an identical lineage, yet distinct functional phenotypes, may compete with each other for space in single-cell-layer tissues. miR-184 expression and secretion were increased in parallel with the cell density, namely with the increased proportion of small-size differentiated HCECs.

Supplementary Information 2

In a previous study by Hamuro and associates [20], the authors investigated the molecular mechanisms of miR-494-3p in extracellular vesicles (EV) released from induced-pluripotent-stem-cell-derived human RPE (iPS-hRPE) and ARPE19 cells differentiated in the presence of nicotinamide (Nic-hRPE). In that study, the molecular interplay of EV miR-494-3p with either mitochondrial selective SIRT3 or organelle nonselective PTEN was found to participate in the degeneration of RPE cells by inducing mitochondrial dysfunctions and repressed OXPHOS, mitochondrial membrane potential, and ATP and NAD^+ production through inactivation of SIRT3 by miR-494-3p. The authors concluded that their results demonstrate a clear causal link between miR-494-3p and RPE cell degeneration via the regulation of mitochondrial integrity (Supplemental Fig. 7, which also appears in [20]).

Recently, paracellular interplay has been widely studied as a new relevant field of cell sociology, and the interplays are divided into two, namely cellular competition and cellular synchronization. In that regard, the findings in the study by Hamuro and associated [20] revealed relevant homotypic cellular competition through EV miR-494-3p among functionally heterogenous (degenerated and non-degenerated) RPE cell subpopulations (SPs) as a pivotal molecule in the aggravated degeneration of RPE cells as well as other cells composed of single-cell layer tissues, as in the CECs illustrated in the previous study by Yamashita and associates [45]. Accordingly, elucidating the

molecular mechanisms that accelerate RPE cell degeneration paracellularly may be critical for understanding AMD pathogenesis.

The plausible interaction of miR-494-3p with PTEN and SIRT3, and the inhibition of the PI3K/Akt pathway by PTEN, are illustrated in Supplemental Figure 7. Activated Akt and SIRT3, both of which are downstream of miR494-3p, enhance the mitochondria functions.

In a previous study by Jiang and associates [41], the authors proposed the direct inactivation of Akt by miR-184 [41], and described that dedifferentiation of RPE cells is a crucial contributing factor to the pathology of retinal degenerative diseases, including AMD. Based on the microarray data, they found that miR-184 was sorted out as the most upregulated signature along with the differentiation from human induced pluripotent stem cells (hiPSC) to RPE cells, thus suggesting its potential promotive role in RPE differentiation. miR-184 promoted RPE differentiation via suppression of AKT2/mammalian target of the rapamycin (mTOR) signaling pathway. AKT2 was upregulated in macular RPE-choroid of the donor with RPE dysfunction and dry AMD patients. Hamuro and associates [20] also proposed the hypothesis that EV miR-184, released from differentiated RPE cells with smaller cell sizes, may participate in the differentiation of differentiation-disposed premature RPE cells (Supplemental Fig. 8).

Supplemental Figure 7

**Competitive Interplay of MiR-494-3p
with Cellular SIRT3 or PTEN
in Coordinating Mitochondria Metabolic Homeostasis**

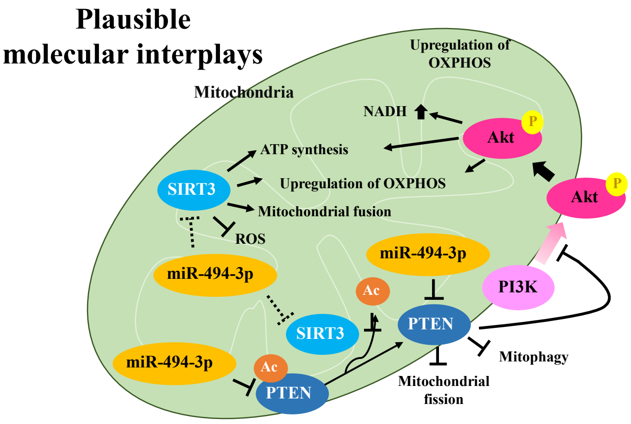

Supplemental Figure 8

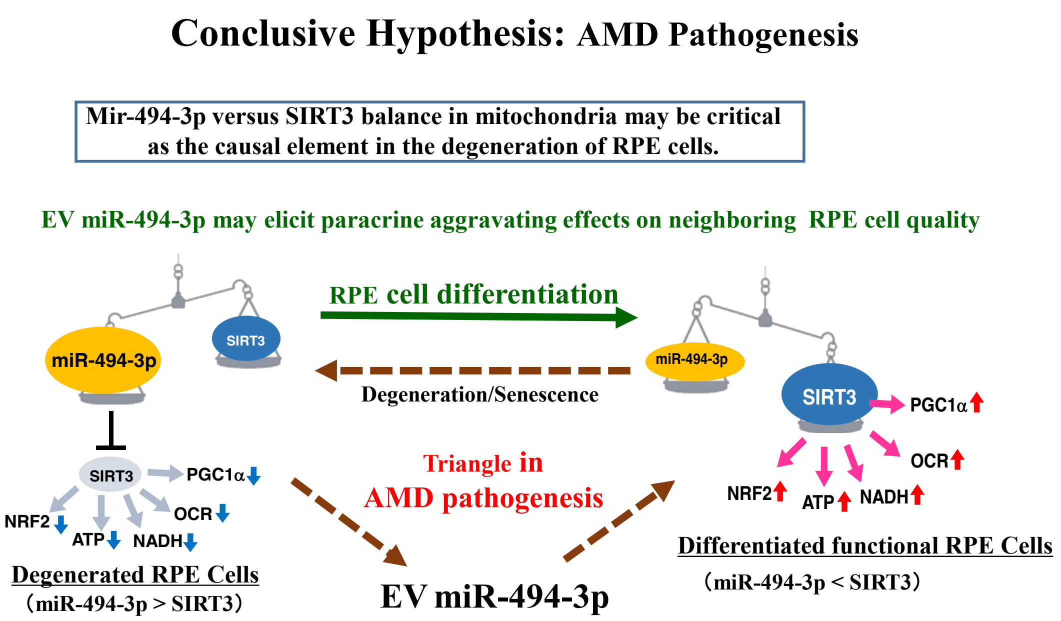

Supplemental Figure 9

Full membrane blots

Figure 2C SIRT1

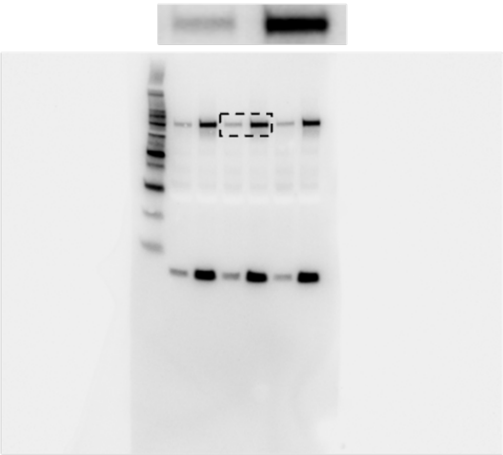

Figure 2C PGC1α

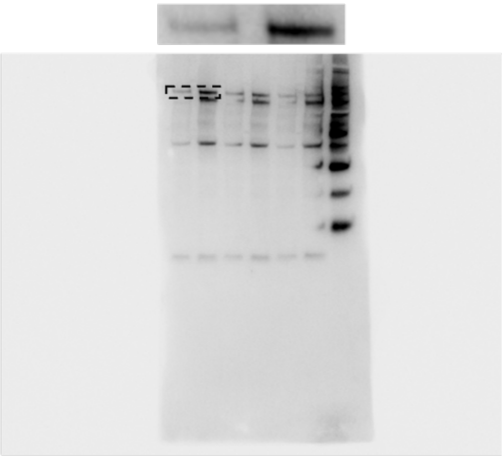

Figure 2C SIRT3

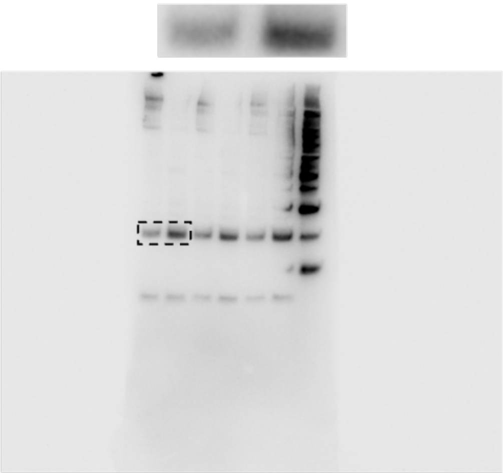

Figure 2C TFAM

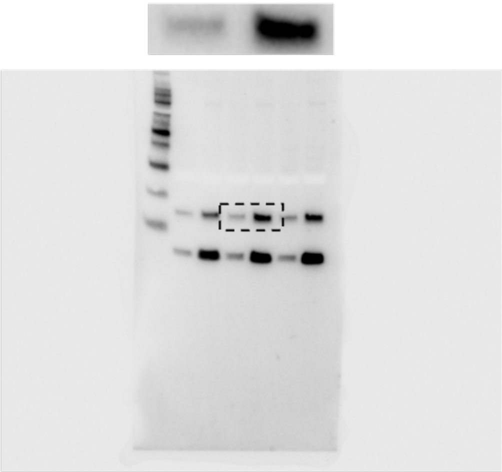

Figure 2C GAPDH,COXIV

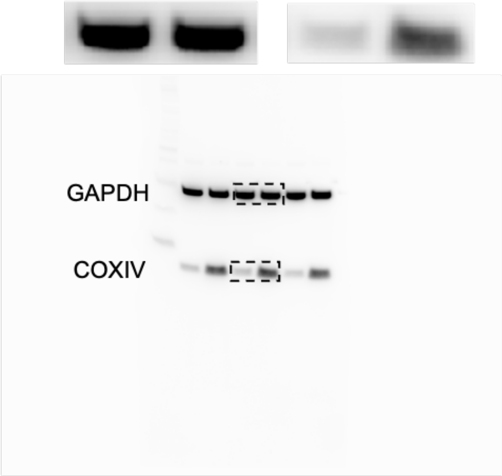

Figure 2C TOMM20

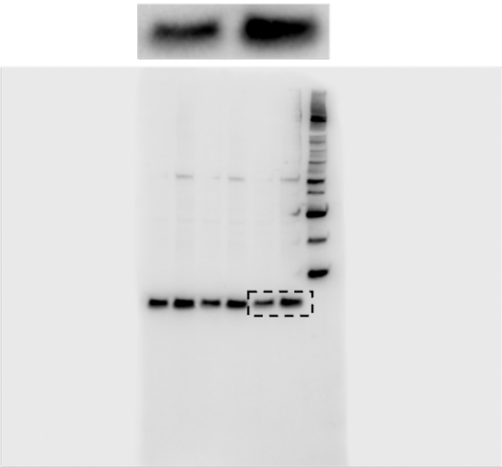

Supplement: Supplementary file 1 — Supplementary Material 1 [file 41598_2024_73145_MOESM1_ESM.pdf]
